# The prevalence of metabolic syndrome among Ghanaian migrants and their homeland counterparts: the Research on Obesity and type 2 Diabetes among African Migrants (RODAM) study

**DOI:** 10.1093/eurpub/ckz051

**Published:** 2019-04-09

**Authors:** Eva L van der Linden, Karlijn Meeks, Erik Beune, Ama de-Graft Aikins, Juliet Addo, Ellis Owusu-Dabo, Frank P Mockenhaupt, Silver Bahendeka, Ina Danquah, Matthias B Schulze, Joachim Spranger, Kerstin Klipstein-Grobusch, Lambert Tetteh Appiah, Liam Smeeth, Karien Stronks, Charles Agyemang

**Affiliations:** 1 Department of Public Health, Amsterdam UMC, University of Amsterdam, Amsterdam Public Health Research Institute, Amsterdam, Netherlands; 2 Regional Institute for Population Studies, University of Ghana, Legon, Ghana; 3 Department of Non-Communicable Disease Epidemiology, London School of Hygiene and Tropical Medicine, London, UK; 4 School of Public Health, Kwame Nkrumah University of Science and Technology, Kumasi, Ghana; 5 Institute of Tropical Medicine and International Health, Charité Universitätsmedizin Berlin, Berlin, Germany; 6 MKPGMS-Uganda Martyrs University, Kampala, Uganda; 7 Institute for Social Medicine, Epidemiology and Health Economics, Charité-Universitätsmedizin Berlin, Berlin, Germany; 8 Department of Molecular Epidemiology, German Institute of Human Nutrition Potsdam-Rehbrücke, Nuthetal, Germany; 9 Department of Endocrinology and Metabolism, Charité Universitätsmedizin Berlin, Berlin, Germany; 10 DZHK (German Centre for Cardiovascular Research), Partner Site Berlin, Berlin, Germany; 11 Center for Cardiovascular Research (CCR), Charité-Universitätsmedizin Berlin, Berlin, Germany; 12 Julius Global Health, Julius Center for Health Sciences and Primary Care, University Medical Center Utrecht, Utrecht University, The Netherlands; 13 Division of Epidemiology and Biostatistics, School of Public Health, Faculty of Health Sciences, University of the Witwatersrand, Johannesburg, South Africa; 14 Department of Medicine, Komfo Anokye Teaching Hospital, Kumasi, Ghana

## Abstract

**Background:**

Metabolic syndrome (MetSyn) is an important risk factor for cardiovascular diseases and type 2 diabetes. It is unknown whether the MetSyn prevalence differs within a homogenous population residing in different settings in Africa and Europe. We therefore assessed the prevalence of MetSyn among Ghanaians living in rural- and urban-Ghana and Ghanaian migrants living in Europe.

**Methods:**

We used data from the cross-sectional multi-centre RODAM study that was conducted among Ghanaian adults aged 25–70 years residing in rural- and urban-Ghana and in London, Amsterdam and Berlin (*n* = 5659). MetSyn was defined according to the 2009 harmonized definition. Geographical locations were compared using age-standardized prevalence rates, and prevalence ratios (PRs), adjusted for age, education, physical activity, and smoking and stratified for sex.

**Results:**

In men, the age-standardized prevalence of MetSyn was 8.3% in rural Ghana and showed a positive gradient through urban Ghana (23.6%, adjusted PR = 1.85, 95% confidence interval 1.17–2.92) to Europe, with the highest prevalence in Amsterdam (31.4%; PR = 4.45, 2.94–6.75). In women, there was a rural-to-urban gradient in age-standardized MetSyn prevalence (rural Ghana 25%, urban Ghana 34.4%, PR = 1.38, 1.13–1.68), but small differences in MetSyn prevalence between urban-Ghanaian and European-Ghanaian women (Amsterdam 38.4%; London 38.2%).

**Conclusion:**

MetSyn is highly prevalent in Ghana as well as in Ghanaian migrants in Europe. To assist prevention efforts, further research is needed to understand the mechanisms driving the geographical differences in MetSyn prevalence between migrant and non-migrant Ghanaians.

## Introduction

Metabolic syndrome (MetSyn) is a collection of five cardiometabolic risk factors including an increased waist circumference (WC), elevated triglyceride levels, lowered high-density lipoprotein cholesterol (HDL-C), elevated blood pressure (BP) and raised fasting plasma glucose (FPG).^1^ Compared with individuals without the syndrome, individuals with MetSyn have a 3- to 5-fold increased risk for developing type 2 diabetes (T2D)^2^^,^^3^ and a doubled risk for cardiovascular disease (CVD) events.^4^ Studies on MetSyn in sub-Saharan African (SSA) countries are scarce, but suggest that MetSyn is of importance in this region, with prevalence rates varying from 13.7% in rural Uganda^5^ to 21% in urban Ghana^6^ and 34.6% in urban Kenya.^7^ Rapid urbanization associated with lifestyle changes have been suggested as a major contributor to this rural–urban difference.^8^ Data on MetSyn prevalence among populations with SSA origin in high-income countries are also limited and show substantial differences in MetSyn prevalence, depending on sex and geographical location of residence.^9^^,^^10^ There is no clear explanation for these differences in MetSyn prevalence, but this might be due to differential exposures in the countries of settlement, leading to variation in distribution and clustering of MetSyn components.^11^ As MetSyn is an important risk factor for CVD and T2D, it is important to obtain more insight into the distribution of MetSyn components among SSA populations and how this varies between populations from SSA origins in different settings. Therefore, the aim of this study was to assess whether the prevalence of MetSyn and its individual components differ among Ghanaian migrants in urban areas in The Netherlands, UK and Germany and in their counterparts living in rural and urban Ghana.

## Methods

The rationale, conceptual framework, design and methodology of the RODAM study have previously been described elsewhere,^12^ and will be summarized here.

### Study population and study design

The RODAM study, a multi-centre, cross-sectional study, was carried out between 2012 and 2015, and included Ghanaians aged ≥25 years living in rural and urban Ghana, and in the cities of London, Amsterdam and Berlin. Ghanaians are one of the largest SSA migrant groups in the UK, Netherlands and Germany, of whom the majority resides in their respective capital cities.^13–15^ Various recruitment strategies were necessary at the different study sites, due to differences in population registration systems across European countries and in Ghana. In brief, in a random sampling procedure, participants were drawn from rural or urban enumeration areas in Ghana, or from municipality registration and Ghanaian organizations in the three European sites. In Ghana, the participation rate was 76% at the rural and 74% at the urban recruitment sites. In Europe, the participation rates were 53% in Amsterdam, 75% in London and 68% in Berlin.

### Data collection, measurements and definitions

Data collection was standardized using standard operation procedures across all sites. Information on demographics, socioeconomic position, migration-related factors, health status and health behaviour was obtained using questionnaires either self-administered or via interviews conducted by trained interviewers. Age showed a normal distribution, and was therefore presented as continuous variable. Education was used as a proxy for socioeconomic position, and was categorized into none or elementary schooling, lower secondary, higher secondary and tertiary education, or unknown level. First generation migrant was defined as being born in Ghana and migrated to Europe. Smoking status was categorized into current or former smoker, or never smoked. The World Health Organization STEPS questionnaire^16^ was used to derive physical activity in metabolic equivalent (MET, hours/week), which included physical activity at work, while commuting and in leisure time.^17^ Answers were subsequently classified based on the guidelines of The IPAQ group,^18^ into low, moderate or high level of physical activity.

Physical examinations were performed with validated devices. Weight and height were measured in light clothing without shoes using the SECA877 weighing scale and SECA217 portable stadiometer. WC was measured using measuring tape at the midpoint between the lower rib and the upper margin of the iliac crest. All anthropometric measurements were performed twice by the same examiner and the average of the two measurements was used for analysis. BP was measured three times after at least 5 min rest, in sitting position with an appropriate cuff, using Microlife WatchBP home, a semi-automated device. The mean of the last two measurements was used in the analysis.

Participants were instructed to fast from 10:00p.m. the night prior to the blood sample collection.

Fasting venous blood samples were collected by trained research assistants. Processing and transportation of the blood samples was standardized across all study sites.^12^ FPG, HDL-cholesterol and triglycerides were determined using the ABX Pentra 400 chemistry analyser (HORIBA ABX, Montpellier, France).

MetSyn was determined using the definition of International Diabetes Federation in collaboration with the National Heart, Lung, and Blood Institute, American Heart Association, World Heart Federation, International Atherosclerosis Society and International Association for the Study of Obesity.^1^ This definition identifies an individual with MetSyn if at least three of the following five criteria are present: elevated WC or abdominal obesity (men ≥94 cm, women ≥80 cm); elevated triglycerides (≥1.7 mmol/l and/or the use of lipid-lowering medication); reduced HDL-cholesterol (men <1.0 mmol/l, women <1.3 mmol/l, and/or the use of lipid-lowering medication); elevated BP (systolic ≥130 and/or diastolic ≥85 mmHg, and/or antihypertensive treatment); raised FPG (≥5.6 mmol/l and/or the use of blood glucose-lowering medication).

### Statistical analysis

Participants aged 25–70 years were eligible for inclusion. 6385 Ghanaians agreed to participate in the study of which 5898 completed physical examination and provided blood samples for biochemical characterization. After exclusion of participants outside the age range, 5659 participants were included in the analysis.

All analyses were stratified by geographical location and sex. Age-standardized prevalence rates of MetSyn and its components were calculated using the age distribution of the total RODAM population as standard population. Prevalence ratios (PRs) with corresponding 95% confidence intervals (CI) were calculated using Poisson regression with model-based variance to compare the prevalence of MetSyn between the study sites.^10^^,^^19^ As the RODAM study conceptualized rural Ghana as the source from where Ghanaians migrated to urban Ghana and subsequently to Europe, we used rural Ghana as the reference category. In the Poisson regression analysis, adjustment was made for covariates in two models. Model 1 was adjusted for age and level of education; model 2 for age, level of education, physical activity and smoking status.^10^ The prevalence rates of MetSyn and its components were compared between the European sites, as this would potentially disclose differences between the populations of the three European study sites. Amsterdam was used as the reference population for the within-Europe comparison, as the population of this study location previously showed to have the highest prevalence of impaired fasting glucose.^19^ The analysis for the European sites was adjusted for the factors included in model 2 plus length of stay in Europe (in years).

Analyses were performed using IBM SPSS Statistics (SPSS Inc., Released 2016. SPSS for Windows, Version 24.0, Chicago, IL, USA). Age-standardized prevalence was calculated using R Statistics (R Core Team. 2017, R for Windows, version 3.4.1. R Foundation for Statistical Computing, Vienna, Austria).

### Ethical approval and consent to participate

Ethical approval of the study was requested from the respective ethics committees in Ghana (School of Medical Sciences/Komfo Anokye Teaching Hospital Committee on Human Research, Publication & Ethical Review Board), the Netherlands (Institutional Review Board of the Academic Medical Center, University of Amsterdam), Germany (Ethics Committee of Charité-Universitätsmedizin Berlin) and the UK (London School of Hygiene and Tropical Medicine Research Ethics Committee) before data collection began in each country. Informed written consent was obtained from each participant prior to the enrolment in the study.

## Results

### Characteristics of study population

A total of 5659 participants were eligible for analysis, of which 62.3% were women. Table 1 shows detailed population characteristics of the included participants. The mean age of the sample was 46.2 years. Amsterdam-Ghanaian men, and London- and rural-Ghanaian women were older compared with participants in other sites. Ghanaians living in London were the most educated population. Ninety-seven percent of the European-Ghanaians were first generation migrants. Smoking prevalence was low in all locations, except among Berlin-Ghanaian men. Ghanaians living in London were least physically active.

**Table 1:** Population characteristics by locality and sex

	Rural-Ghanaians	Urban-Ghanaians	Amsterdam-Ghanaians	Berlin-Ghanaians	London-Ghanaians
Men, *n* (%)	405 (19.0)	415 (19.4)	609 (28.5)	297 (13.9)	410 (19.2)
Age, years (CI)	46.2 (45.0–47.5)	46.5 (45.4–47.7)	48.4 (47.7–49.2)	45.8 (44.5–47.1)	46.1 (45.0–47.1)
Education level, % (CI)					
None or elementary	39.0 (34.4–43.8)	22.2 (18.4–26.3)	20.5 (17.5–23.9)	6.1 (3.8–9.2)	3.9 (2.3–6.1)
Lower secondary	36.0 (31.5–40.8)	42.4 (37.7–47.2)	40.6 (36.7–44.5)	47.8 (42.2–53.5)	24.9 (20.9–29.2)
Higher secondary	13.3 (10.3–16.9)	20.5 (16.8–24.6)	25.1 (21.8–28.7)	28.3 (23.4–33.6)	16.8 (13.4–20.7)
Tertiary	5.7 (3.7–8.3)	9.2 (6.7–12.2)	8.2 (6.2–10.6)	17.5 (13.5–22.1)	41.0 (36.3–45.8)
Unknown	5.9 (3.9–8.5)	5.8 (3.8–8.3)	5.6 (4.0–7.6)	0.3 (0.0–1.6)	13.4 (10.4–17.0)
Duration of stay in Europe, years (CI)	n/a	n/a	18.7 (18.0–19.4)	16.8 (15.5–18.2)	15.1 (14.1–16.1)
First generation migrant, % (CI)	n/a	n/a	98.6 (97.3–99.3)	99.3 (97.9–99.9)	98.6 (97.0–99.5)
Current smoking, yes, % (CI)	5.8 (3.8–8.5)	3.3 (1.9–5.4)	8.1 (6.1–10.6)	14.8 (11.1–19.2)	1.3 (0.5–2.9)
Physical activity, % (CI)					
Low level	10.8 (7.9–14.2)	22.4 (18.5–26.7)	20.6 (16.8–24.8)	24.2 (19.6–29.3)	41.4 (36.5–46.5)
Moderate level	16.5 (13.1–20.5)	18.6 (15.0–22.6)	14.5 (11.3–18.2)	20.9 (16.5–25.8)	18.3 (14.6–22.4)
High level	71.9 (67.3–76.3)	57.5 (52.6–62.3)	59.3 (54.4–64.1)	52.2 (46.5–57.8)	31.2 (26.6–36.0)
Women, *n* (%)	638 (18.1)	1034 (29.3)	931 (26.4)	250 (7.1)	670 (19.0)
Age, years (CI)	46.7 (45.7–47.6)	44.7 (44.0–45.4)	45.6 (45.0–46.1)	44.7 (43.5–45.9)	47.7 (46.9–48.5)
Education level, % (CI)					
None or elementary	62.2 (58.4–65.9)	50.5 (47.4–53.5)	40.9 (37.7–44.0)	11.6 (8.1–16.0)	10.0 (7.9–12.4)
Lower secondary	25.9 (22.6–29.4)	35.9 (33.0–38.8)	30.6 (27.7–33.7)	54.0 (47.8–60.1)	29.9 (26.5–33.4)
Higher secondary	3.0 (1.9–4.5)	8.5 (6.9–10.3)	17.8 (15.5–20.4)	24.8 (19.8–30.4)	24.2 (21.1–27.5)
Tertiary	1.9 (1.0–3.2)	2.7 (1.8–3.8)	3.8 (2.7–5.1)	7.6 (4.8–11.4)	22.1 (19.1–25.3)
Unknown	7.1 (5.3–9.2)	2.4 (1.6–3.5)	6.9 (5.4–8.6)	2.0 (0.8–4.3)	13.9 (11.4–16.7)
Duration of stay in Europe, years (CI)	n/a	n/a	17.7 (17.2–18.2)	16.9 (15.7–18.2)	17.4 (16.5–18.3)
First generation migrant, % (CI)	n/a	n/a	99.5 (98.9–99.8)	99.6 (98.1–100.0)	96.9 (95.3–98.1)
Current smoking, yes, % (CI)	0 (0.0)	0.1 (0.0–0.5)	2.1 (1.3–3.2)	3.3 (1.5–6.0)	0.2 (0.0–0.8)
Physical activity, % (CI)					
Low level	23.2 (20.0–26.8)	40.4 (37.4–43.4)	16.5 (13.8–19.5)	31.3 (25.8–37.3)	41.6 (37.7–45.5)
Moderate level	23.1 (19.8–26.6)	15.7 (13.6–18.1)	21.8 (18.8–25.1)	18.3 (13.8–23.5)	23.8 (20.6–27.3)
High level	53.5 (49.5–57.5)	43.2 (40.2–46.3)	55.8 (52.0–59.6)	45.5 (39.4–51.8)	24.8 (21.5–28.3)

Notes: Values are means or percentages with corresponding 95% confidence intervals. *n*, number; n/a, not available; CI, confidence interval.

### Prevalence of MetSyn

**Figure 1:**
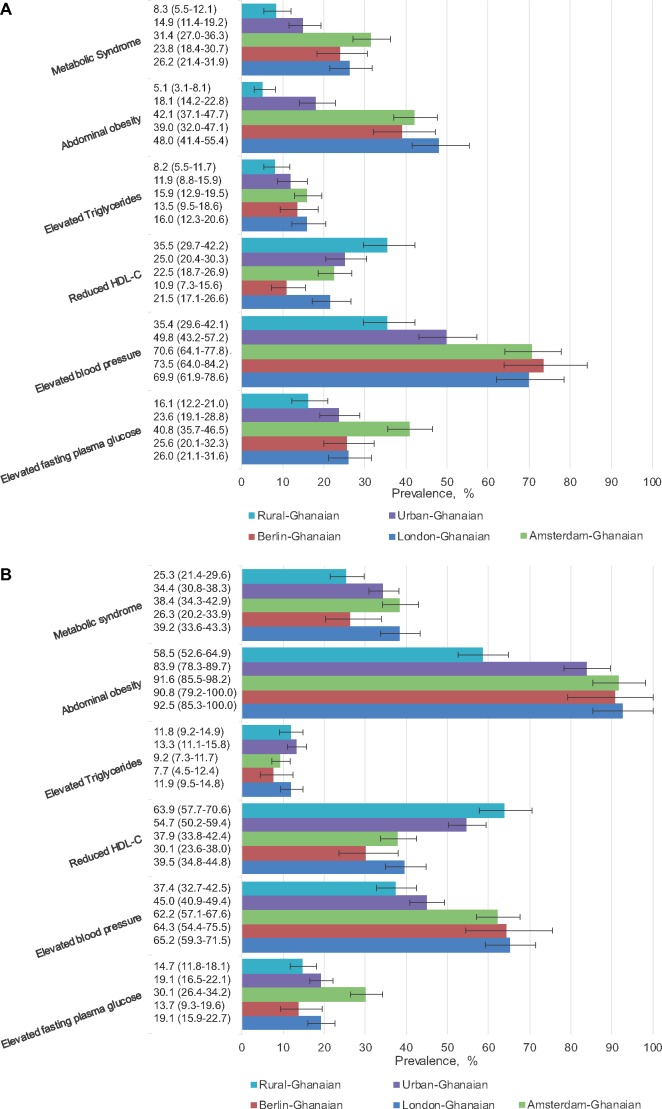
Age-standardized prevalence of metabolic syndrome, abdominal obesity, elevated triglycerides, reduced high-density lipoprotein cholesterol (HDL-C), elevated blood pressure and elevated fasting plasma glucose, by site in men (A) and women (B). Error bars are 95% confidence intervals

**Figure 2:**
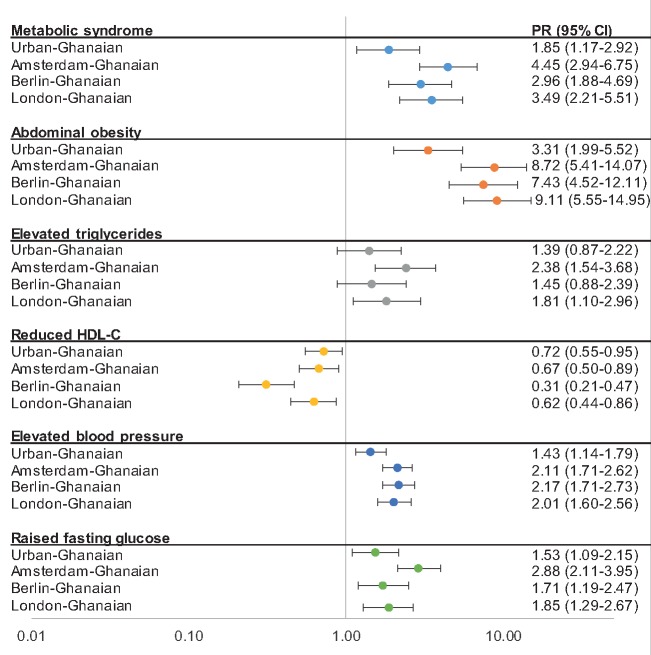
Prevalence ratios (PRs) for metabolic syndrome and its components for men by site, compared with rural Ghana (reference population, PR = 1.00). Error bars are 95% confidence intervals. Model adjusted for age, level of education, physical activity and smoking status

**Figure 3:**
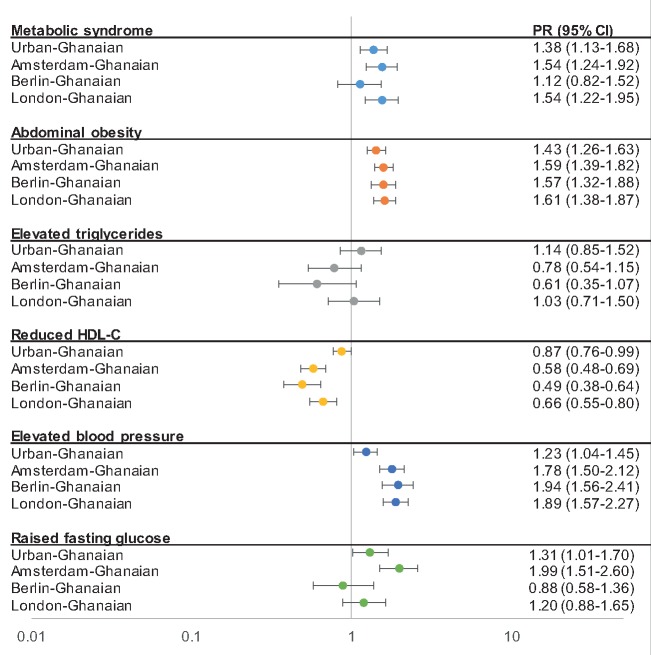
Prevalence ratios for metabolic syndrome and its components for women by site, compared with rural Ghana (reference population, PR = 1.00). Error bars are 95% confidence intervals. Model adjusted for age, level of education, physical activity and smoking status

In men, the age-standardized MetSyn prevalence ranged from 8% (95% CI, 5.5–12.1) among rural-Ghanaians to 31% (27.0–36.3) among Amsterdam-Ghanaians, with a positive gradient in prevalence through urban Ghana to European sites (figure 1A). This positive gradient was also observed between rural- and urban-Ghanaian women [rural Ghana 25% (21.4–29.6), urban Ghana 34% (30.8–38.3)], but not between urban-Ghanaian and European-Ghanaian women (figure 1B). The adjusted PR of MetSyn was almost two times higher for urban-Ghanaian men compared with rural-Ghanaian men, and increased to a more than four times higher PR in Amsterdam-Ghanaian men (figure 2). Among women, PRs did not vary greatly between the sites (figure 3), except for Berlin-Ghanaian women, who had a lower PR compared with the urban-Ghanaian and the other European sites. Comparison between the European sites, with additional adjustment for length of stay in Europe, strengthened the trend towards a lower MetSyn PR in Berlin- compared with Amsterdam-Ghanaians (Supplementary figure S1).

### Prevalence of MetSyn components

As the PRs for the MetSyn components did not vary between model 1 and model 2, only the results for model 2 are presented.

#### Abdominal obesity

In men, the age-standardized prevalence of abdominal obesity showed a positive rural–urban-Europe gradient (figure 1A). In women, abdominal obesity was highly prevalent at all sites, and showed a prevalence gradient between rural and urban Ghana, but not between urban Ghana and Europe (figure 1B). This was reflected in the PRs, which showed a positive rural–urban-Europe gradient in men (figure 2), but no difference between urban Ghana and Europe in women, compared with rural Ghana (figure 3). Moreover, the PRs did not differ between the European sites (Supplementary figure S1).

#### Elevated triglycerides

In men, the age-adjusted prevalence of elevated triglycerides did not vary between the locations, but a trend towards a higher prevalence in urban- and European-Ghanaians could be observed (figure 1A). In women, the prevalence tended to be higher in rural- and urban-Ghanaian women than in European-Ghanaian women (figure 1B).

Compared with rural-Ghanaian men, the adjusted PRs were twice as high in Amsterdam- and London-Ghanaian men, and comparable with urban- and Berlin-Ghanaian men. This was also reflected in the comparison of PRs between the European sites, which showed a lower PR for elevated triglycerides in Berlin- compared with Amsterdam-Ghanaian men (Supplementary Figure S1). In women, the PRs for elevated triglycerides were similar between study sites, with a trend towards lower PRs in Amsterdam and Berlin compared with rural Ghana (figure 3).

#### Reduced HDL-C

The age-adjusted prevalence of reduced HDL-C showed a negative gradient from rural Ghana, through urban Ghana to Europe, in both sexes, with the lowest prevalence of reduced HDL-C in Berlin-Ghanaians (figure 1A and B ). The adjusted PRs followed a similar pattern, with especially low PRs in Berlin- compared with rural-Ghanaian men and women (figures 2 and 3). Within the European sites, Berlin-Ghanaian men had a lower PR for reduced HDL-C compared with Amsterdam-Ghanaian men (Supplementary figure S1).

#### Elevated blood pressure

An elevated BP was more prevalent in Ghanaian migrants than participants residing in Ghana (figure 1A and B). The adjusted PRs showed a positive rural-urban-Europe gradient, with the PR being twice as high in the European sites compared with rural Ghana (figures 2 and 3). There were no differences in PRs between the European sites.

#### Raised fasting plasma glucose

In men, the age-adjusted prevalence and adjusted PRs of raised FPG showed a positive rural-to-urban gradient, with resembling prevalence between urban Ghana and Europe. In women, the prevalence rates were comparable between urban Ghana and London, and the adjusted PRs did not differ between rural Ghana, Berlin and London. In Europe, Amsterdam-Ghanaians had the highest prevalence of raised FPG (figure 1A and B), which was reflected in the PRs (Supplementary figure S1).

## Discussion

### Key findings

The MetSyn prevalence among Ghanaians differed by locality. In men, the MetSyn prevalence showed a positive gradient from rural Ghana, through urban Ghana to Europe, with a higher MetSyn prevalence in Amsterdam-Ghanaian men. In women, there was a positive gradient in MetSyn prevalence between rural- and urban-Ghanaian women. However, this prevalence did not differ between urban-Ghanaian women compared with their European-Ghanaian counterparts.

The prevalence rates of the individual MetSyn components—except for reduced HDL-C—were higher in urban Ghana compared with rural Ghana, and even higher in Europe. A decreased HDL-C level was more prevalent among rural-Ghanaians compared with their urban and European counterparts. There was a high prevalence of raised FPG in Amsterdam-Ghanaian men and women, which substantially accounted for the lower MetSyn prevalence in Berlin-Ghanaians compared with Amsterdam-Ghanaians.

### Discussion of the key findings

The MetSyn prevalence showed a positive rural-to-urban gradient in both sexes, which seems to suggest that exposure to an urban environment, potentiates the development of metabolic risk factors.^20^ This is in line with findings from other SSA studies.^21–23^ Moreover, MetSyn occurred at nearly the same rate among urban-Ghanaian women compared with their European-Ghanaian counterparts, suggesting that similar underlying processes might be at play in urban areas in Ghana as in migrant populations in Europe.

The MetSyn prevalence is higher in Ghanaian migrant women than in women from the respective European host populations, whereas MetSyn is less prevalent in Ghanaian men compared with European men.^11^^,^^24^^,^^25^ These results are in line with findings from the USA, showing men from ethnic minorities being less affected by MetSyn, whereas women from ethnic minorities being more affected by MetSyn compared with the European descent population.^26^ The differences in MetSyn prevalence rates between the migrant and European host population is mostly attributable to the high prevalence of abdominal obesity in Ghanaian women.^10^^,^^11^^,^^21^^,^^27^ Genetic predisposition, differential exposures in early life and gene-environmental interactions might contribute to this disease susceptibility.^12^ Moreover, sociocultural perceptions regarding ideal body size may also contribute to the high abdominal obesity rate in women.^28^

The prevalence of raised FPG was remarkably higher among Amsterdam-Ghanaian migrants compared with Berlin- and London-Ghanaians and seems to be the major driving factor behind the differences in MetSyn prevalence between these European sites. These findings are in line with a previous study, showing a raised FPG prevalence of 35 and 14% in non-diabetic Amsterdam-African Caribbean’s and London-African Caribbean’s respectively, despite a higher prevalence of central obesity in the latter.^10^ The reason for the high susceptibility of Amsterdam-Ghanaian migrants for having raised FPG is not clear, but might be due to differences in contextual factors, such as differences in access to health care and preventive services,^29^ psychosocial factors^30^ or dietary habits. Moreover, these contextual factors could initiate epigenetic modifications, thereby shaping the glucose metabolism differently among similar populations living in different countries. Therefore, further research is required to clarify these intercountry variations.

Another interesting finding of our study is the high prevalence of reduced HDL-C in rural-Ghanaian residents, which showed to be significantly higher in this group compared with the prevalence in urban-Ghanaian and European-Ghanaian residents. These findings are in line with previous reports from Ghana and other SSA countries, demonstrating high prevalence rates of reduced HDL-C in both rural and urban populations, with higher prevalence rates in women than in men.^7^^,^^23^^,^^31^ These low levels of HDL-C in rural Ghana might be due to specific dietary habits, which appear to differ from the other geographical locations as previous RODAM results showed.^32^ As HDL-C has a key role in the transport of excessive cholesterol towards the liver for excretion, and has anti-thrombotic and anti-inflammatory properties, it fulfils an important cardioprotective role.^33^ Therefore, the high prevalence of reduced HDL-C in the Ghanaian population points to a potential important CVD risk factor and needs further research.

### Strengths and limitations

This study is the first to look at MetSyn in a homogenous SSA migrant population in comparison to the population in their home country in SSA. Previous studies used migration surrogates (e.g. comparing native Africans with African-Americans) to study the effect of migration on CVD risk factors. However, people from the African diaspora originate from heterogeneous ancestry and show high levels of genetic diversity,^34^ which hampers the comparison with populations residing in SSA. Moreover, none of these migration studies focussed on MetSyn. The use of highly standardized operating procedures for data collection across all study sites made it possible to compare the different geographical locations. However, as the RODAM study has a cross-sectional study design, the possibilities to study the causality between MetSyn and the determinants tested in the statistical models are limited. In addition, as recruitment strategies had to be tailored to the different study locations, a certain selection bias might have occurred. However, as the non-response analysis revealed similar patterns between all sites, we consider it unlikely that the differences in prevalence rates between the sites are biased by the variations in sampling strategy. As we performed full case analysis, participants with missing data on education, smoking or physical activity were excluded from the Poisson regression analysis. However, the participants excluded from analysis did not differ from those included, in terms of age or sex. In addition, those with missing data did not differ from those with complete data with regards to prevalence of MetSyn or its components. Therefore, we do not expect missing data on these covariates to influence our results in a significant way. Lastly, different methods were used to administer the questionnaire, which could introduce some level of bias as varied methods could have influenced the participants’ answers differently. However, only small percentage of Ghanaian migrants in Europe (12%) self-administered the questionnaire. In addition, as research assistants were trained to conduct the interviews in a standardized way, we consider this limitation to be a minor source of bias.

## Conclusion

Our findings show a positive gradient of the MetSyn prevalence from rural Ghana, through urban Ghana to Europe but only in men. The prevalence rates of the individual MetSyn components (abdominal obesity, elevated triglyceride, elevated BP and raised FPG) generally show positive gradients whereas reduced HDL-C shows a negative gradient from rural Ghana, through urban Ghana to Europe. In addition, the prevalence of MetSyn and its components vary among Ghanaian migrants living in different European countries. These distinctive geographical differences between the groups show the potential importance of contextual factors on cardiometabolic risk and suggest the need to unravel the underlying mechanisms to guide prevention management efforts in SSA populations.

## Supplementary Material

ckz051_Supplementary_Figure
