# Supplementary figures and images for: The prevalence of metabolic syndrome among Ghanaian migrants and their homeland counterparts: the Research on Obesity and type 2 Diabetes among African Migrants (RODAM) study
Source: Eur J Public Health. 2019 Apr 9;29(5):906–13. doi: 10.1093/eurpub/ckz051 (PMC6761842; doi:10.1093/eurpub/ckz051)

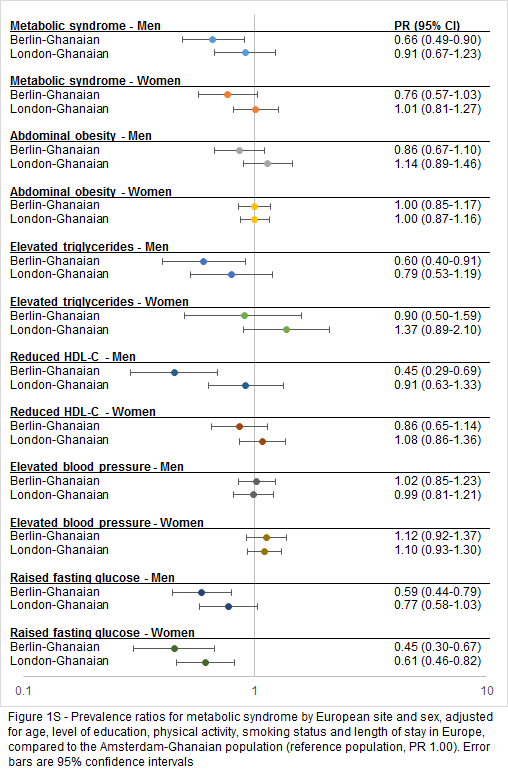

Supplement: ckz051_Supplementary_Figure [file ckz051_supplementary_figure.png]
